# Ophthalmic and Oculomotor Characteristics in Ataxia-Telangiectasia: A Clinical Cohort Study using Video-Oculography

**DOI:** 10.1007/s44402-026-00075-7

**Published:** 2026-04-08

**Authors:** Ann L. Webber, Larry Abel, Heidi Rose Neilson, David Coman, Martin Lavin, Matt Lynch, Robert S. Ware, Shuan Dai

**Affiliations:** 1 Department of Ophthalmology, Queensland Children’s Hospital, Brisbane, Australia; 2 Centre for Vision and Eye Research, Faculty of Health, Queensland University of Technology, Brisbane, Queensland Australia; 3 School of Medicine (Optometry), Deakin University, Waurn Ponds, Victoria Australia; 4 Department of Metabolic Medicine, Queensland Children’s Hospital, Brisbane, Australia; 5 School of Medicine, University of Queensland, Brisbane, Australia; 6 School of Medicine, Griffith University, Gold Coast, Australia; 7 Wesley Research Institute, Brisbane, Australia

**Keywords:** Ataxia-Telangiectasia, Nystagmus, Pursuits, Saccades, Video-oculography, Visual acuity

## Abstract

**Purpose:**

To characterise the ophthalmic and oculomotor features of Ataxia-Telangiectasia (A-T) and to evaluate the feasibility and utility of video oculography as part of a comprehensive visual assessment.

**Methods:**

Thirty participants aged 3 to 36 years (15.7 $\pm$ 9.8 years) with genetically confirmed A-T underwent detailed ophthalmic evaluation, including visual acuity, refraction, binocular function, slit-lamp and fundus examination, including optical coherence tomography. Non-invasive eye movement recordings of self-paced and reflexive saccades, smooth pursuit and gaze-holding in horizontal and vertical planes were made using the EyeSeeCam video-oculography (VOG) system.

**Results:**

Visual acuity was preserved in most patients, with 24 (79%) achieving 6/12 or better. Refractive errors were present in 15 (50%), predominantly low hyperopia or myopia. Strabismus was identified in four (13%) and moderate to severe conjunctival telangiectasia was present in 20 (67%) patients. Eye movement recordings revealed hypometric saccades, increased latency, impaired smooth pursuit (especially vertical) and frequent nystagmus. Self-paced saccades emerged as the most reliable and interpretable oculomotor measure, with counts over a 30-s period ranging from 8 to 58 (median = 20), reflecting significant variability in volitional eye movement control.

**Conclusions:**

Patients with A-T exhibit a spectrum of ophthalmic abnormalities, with significant oculomotor dysfunction despite relatively preserved acuity. Self-paced saccade metrics derived from VOG recordings offer a robust and interpretable measure of oculomotor function, and may serve as adjunctive biomarkers for disease monitoring and treatment evaluation. The integration of VOG into clinical assessment provides qualitative and quantitative insights into eye movement control in A-T.

Key Points

- This study shows that people with ataxia telangiectasia frequently have preserved visual acuity, despite significant difficulties initiating and controlling eye movements, highlighting the value of detailed assessment to understand functional visual difficulties.
- Self-paced saccade counts emerged as reliable indicators of neurological involvement, suggesting their potential use as practical, quantitative measures for monitoring changes in disease severity or response to treatment.
- A simple bedside task for counting voluntary eye movements provides a practical alternative when specialised video systems are unavailable and supports earlier recognition of functional challenges within routine clinical care.

## Introduction

Ataxia-Telangiectasia (A-T) is an autosomal recessive multisystem disorder with an estimated incidence of 1 to 3:1,000,000 births [1, 2]. A-T is listed in the Online Mendelian Inheritance in Man database under entry OMIM 208900, which denotes the classic A‑T phenotype resulting from pathogenic variants in the ATM gene. The condition is characterised by progressive cerebellar degeneration, immunodeficiency, cancer susceptibility and pulmonary complications [3–6]. Neurological manifestations, including impaired gait, hand incoordination and abnormal eye movements, are among the earliest and most functionally disabling features of the condition [7].

Ocular involvement in A-T is well documented, with conjunctival telangiectasia serving as a hallmark feature. However, the broader ophthalmic phenotype, impacting visual acuity, refractive error, binocular function and structural integrity of ocular tissues, has not been characterised systematically in a clinical cohort. Previous studies have focused primarily on oculomotor abnormalities such as oculomotor apraxia, nystagmus and hypometric saccades, often using invasive or technically limited methods that do not account for head movement or fixation instability [8–12]. In contrast, the present study employed a non-invasive video-oculography (VOG) system capable of capturing eye movements while accounting for head movement, enabling a more detailed and objective assessment of oculomotor function than traditional grading scales, and situated within the context of a comprehensive ophthalmic clinical examination.

This study aimed to provide a comprehensive report of ophthalmic findings in individuals with A-T, integrating visual function, ocular health and oculomotor analysis. By embedding eye movement recordings within a broader clinical assessment, the study sought to contextualise oculomotor deficits alongside other visual impairments and explore their potential relevance to disease severity and functional outcomes. It also aimed to assess the feasibility and reliability of VOG-derived oculomotor metrics as potential quantitative outcome measures for therapeutic trials in A-T. These findings may inform clinical care, support educational accommodations and contribute to the development of biomarkers for therapeutic trials.

## Methods

### Study Design and Setting

The clinical cohort study was undertaken in Brisbane, Australia, between February 2022 and April 2023. This study was conducted as part of a broader clinical research programme embedded within a Phase 2a/b randomised, placebo-controlled, dose-escalation trial investigating triheptanoin for the treatment of mitochondrial dysfunction in Ataxia-Telangiectasia: Treating Mitochondrial Dysfunction with a Novel Form of Anaplerosis (A-TC7) (NCT045130020) study registration date 14 August 2020)] [13].

These assessments were performed between February 2022 and April 2023 at the Queensland Children’s Hospital Ophthalmology Clinic, Brisbane, Australia. The study was approved by the human research ethics committees of Children’s Health Queensland (HREC/20/QCHQ/67257), UnitingCare Health (2017) and University of Queensland (2023/HE002409).

### Participants

The National A-T Clinic in Brisbane is a multidisciplinary service based at the Wesley Medical Research Centre and the Queensland Children’s Hospital, providing coordinated specialist care across neurology, immunology, respiratory medicine, genetics, ophthalmology, dermatology and allied health for patients with ataxia-telangiectasia across Australia. Recruitment for the trial was offered to all Australian patients known to the National A-T Clinic who had confirmed pathogenic variants in the *ATM* gene (OMIM 607585). Eligible participants were identified through clinic records. To avoid any clinician–patient power imbalance, treating clinicians did not approach families directly; instead, the study nurse/coordinator sent invitations and study information via mail and email, followed by a phone call approximately 2 weeks later. No incentives were offered.

Individuals of any age and sex with a confirmed diagnosis of A-T were eligible for participation if they were able to undertake the study procedures. Inclusion also required that families were able to comply with the study protocol for its 12-month duration. Written informed consent (and assent where appropriate) was obtained from all participants or their parents/legal guardians, or adult participants in accordance with local regulations. Ophthalmic assessments reported in this manuscript were conducted independently of treatment allocation.

### Ophthalmic Assessment Protocol

Comprehensive ophthalmic evaluation included assessment of visual acuity, ocular alignment and motility and ocular health. Visual acuity was assessed using age-appropriate logMAR LEA symbols or Early Treatment of Diabetic Retinopathy Study letters presented on a rear-illuminated digital screen at 4 m. All participants wore their corrective spectacles, if needed, and a Spielmann Translucent Occluder [14] was used for those with nystagmus. No time restrictions were imposed, as there should not be a penalty for children with nystagmus. The Ishihara test was employed for colour vision and cycloplegic retinoscopy and autorefraction for refractive error. Ocular alignment was assessed by cover tests at distance and near fixation. Clinical grading of pursuits, saccades and observed nystagmus was determined using the oculomotor subscale of the International Cooperative Ataxia Rating Scale (ICARS) [15]. Ocular health was examined via slit-lamp biomicroscopy and anterior segment photography, fundus photography and optical coherence tomography (OCT), when possible, to assess the retinal nerve fibre layer and optic nerve head morphology. While all participants attempted the complete battery of ophthalmic and oculomotor assessments, reliable data were not obtained for all individuals on each test owing to limitations in task comprehension, attentional capacity or fixation stability. Results are reported for successfully interpretable outcomes only; therefore, the number of participants varies across assessments and is indicated as appropriate.

### Eye Movement Recording

Eye movements were recorded by VOG using the EyeSeeCam system (EyeSeeTec GmbH; eyeseetec.de) and software (Interacoustics A/S, version r3523; interacoustics.com). The system employs a monocular, non-invasive, goggle-mounted infrared camera that was positioned before the right eye for all recordings. The system sampled at 220 Hz and included an inertial measurement unit to track head movement. All tests were performed with standard overhead fluorescent room lighting turned on. The EyeSeeCam system provides its own infrared illumination for eye tracking and does not require reduced ambient lighting.

Targets were displayed on a monitor viewed at a 60 cm distance, with the head stabilised. A chin rest was used to minimise head movement during recordings for older children and adults. Younger children were seated on a parent’s lap, with the parent instructed to stabilise the child’s head gently throughout the procedure. Calibration to screen-presented targets was performed prior to test recording. If standard calibration was invalid, then the system’s default calibration was used. Total recording time was under 10 min, with rest breaks as needed. Each recording was matched to patient identifiers and session metadata.

Participants completed a brief oculomotor protocol that comprised self-paced and reflexive saccades, smooth pursuit and gaze-holding in horizontal and vertical planes based upon the protocol used in a cross-sectional oculomotor study of Niemann–Pick disease type C [16]. All visual stimuli were 1° cartoon images (Minion or Peppa Pig emojis, 1 cm diameter), chosen to enhance engagement for children. For the self-paced saccade task, participants alternated their gaze between two continuously present targets at ±15° for 30 s. In the reflexive saccade tasks, horizontal (H) targets were presented at ±15° and ±30°, while vertical (V) targets were presented at ±10° and ±20°. Fourteen 15° (H) or 10° (V) and eleven 30° (H) or 20° (V) presentations were delivered at 3.33-s intervals with randomised directions; the central target was not present during the 30° (H) or 20° (V) jumps. Targets appeared for 4 s at each location. Smooth pursuit consisted of three cycles of a 0.2 Hz sinusoidal trajectory at ±15° amplitude in both horizontal and vertical planes, with velocity varying sinusoidally and no pauses at the extremes, for a duration of 15 s. For gaze-holding, the target was presented at each of four eccentric positions, 20° right, 20°left, 10° up and 10° down, with recording made for 10 s in each position.

**Fig. 1:**
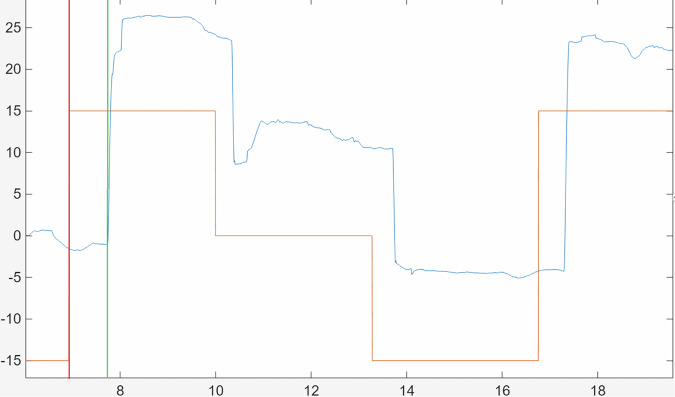
Example reflex saccades plots derived from the EyeSeeCam raw data. Orange lines denote the target position in degrees. The blue lines represent eye position recordings. Latency was calculated from the time difference between the target position change (red vertical line) to first response eye position change (green vertical line). The *x*- and *y*-axes are time in seconds and eye position in degrees, respectively.

Eye movement recordings were analysed using a combination of visual inspection of plots generated by the EyeSeeCam system and quantitative metrics from exported raw data. Due to frequent nystagmus, inconsistent fixation, poor calibration and fragmented signal quality, particularly in younger children and patients with ataxia, quantitative analysis was deliberately restricted. Recordings were reviewed manually for interpretability using an interactive MATLAB graphical interface (mathworks.com), with assessments based on trace quality and calibration integrity. Self-paced saccades were recorded as the number of saccades in 30 s. Reflexive saccade latency was measured directly from the raw, unfiltered position traces, and only when the investigator could clearly identify saccadic onset as shown in Fig. 1. No automated filtering, blink detection or interpolation was applied; trials with data loss were excluded. While the system provides velocity outputs for reflexive saccades, these were not analysed, as calibration instability and nystagmus produced unreliable velocity estimates. Similarly, calibration limitations and prevalent nystagmus prevented reliable pursuit gain and peak‑velocity calculations, as nystagmus oscillations distort measured amplitudes and can lead to misclassification of fast‑phase movements as part of the saccade. Pursuit was assessed descriptively, and pursuit gain could not be derived. Slow-phase velocity during gaze-holding was extracted where possible, but interpreted with caution due to variable trace quality.

### Data Management and Statistical Analysis

Standardised clinical data recording sheets were used to ensure consistency. All data were de-identified and stored securely in accordance with ethical guidelines. Descriptive statistics were used to summarise ophthalmic and eye movement parameters as frequency and percentage for categorical variables and either mean and standard deviation or median and mean absolute deviation, depending on distribution, for continuous variables.

## Results

### Participants

Thirty participants were enroled, ranging from 3 to 36 years of age (mean age = 15.7 $\pm$ 9.8 years); 18 (60%) were female.

### Visual Acuity, Refractive Error, Ocular Alignment and Colour Vision

**Fig. 2:**
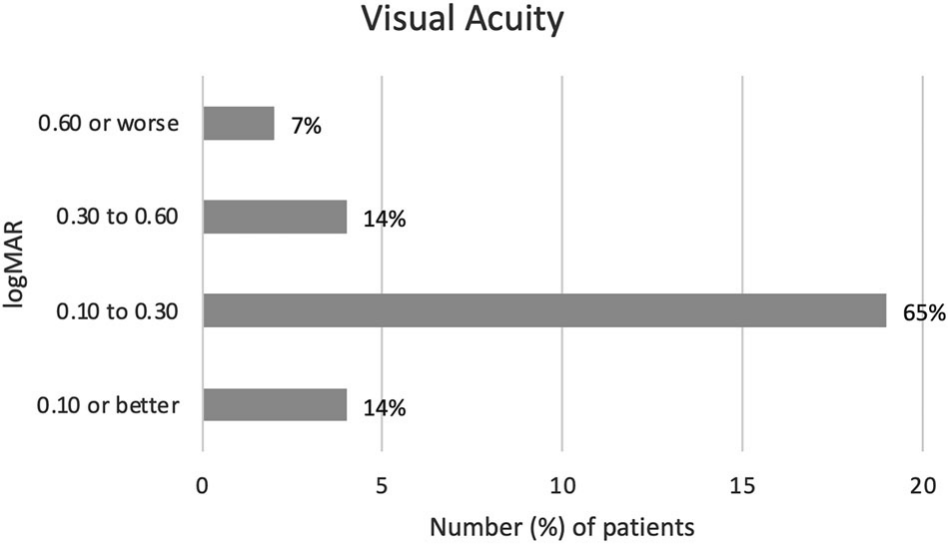
Distribution of visual acuity among the 29 participants who completed quantifiable testing. Visual Acuity (VA) categories follow logMAR cut-points relevant to near-normal, mild and moderate visual reduction.

Visual acuity ranged from normal to significant impairment. Fix and follow visual behaviour was noted in one 3-year-old child who was unable to perform the optotype matching acuity assessment reliably. Among the 29 participants who completed quantifiable acuity testing, four participants (14%) achieved 0.10 logMAR in their better-seeing eye, 19 participants (65%) achieved between 0.10 and 0.30 logMAR, four (14%) had acuity between 0.30 and 0.60 logMAR and two (7%) had acuity worse than 0.60 logMAR. The distribution of Visual Acuity (VA) is shown in Fig. 2. Significant refractive error was present in 11 participants (36%). Based on the spherical equivalent of the more ametropic eye, six participants demonstrated low myopia (-0.50 to –3.00 D), two demonstrated low hyperopia (+1.00 to +3.00 D), two had more than 3.00 D of myopia, and one participant demonstrated hyperopia >+3.00 D. Three had anisometropic amblyopia. No colour vision abnormalities were detected. Strabismus was identified in 13% (4/30), with two cases of esotropia (16 Δ constant esotropia; 6 Δ intermittent esotropia) and two of exotropia (10 Δ constant exotropia; 10 Δ intermittent exotropia).

### Ocular Structures

Clinical slit-lamp photographs were obtained for 27 participants. A descriptive grading approach was used to assess the extent of conjunctival telangiectasia, based on vessel dilation and the degree of scleral obscuration. Conjunctival telangiectasia was graded descriptively as mild when ≤2 vessels showed significant dilation; moderate when >2 vessels were dilated and less than half of the underlying sclera was obscured and severe when >2 vessels were dilated and more than half of the sclera was obscured. Based on this descriptive scale, telangiectasia was graded as mild, moderate and severe in 11 (41%), nine (33%) and seven (26%) participants, respectively. Anterior segment health was otherwise unremarkable.

Posterior segment imaging using OCT was completed successfully in 21 participants, with imaging limited due to fixation instability. Of these, 20 scans (95%) showed a normal macular and optic nerve retinal nerve fibre layer profile; one patient exhibited non-specific maculopathy.

### Eye Movement Characteristics

#### Clinical Observations

Clinical oculomotor examination was recorded in 27 patients. Anomalies recorded included hypometric saccades, vertical (both upbeat and downbeat) and horizontal primary position and gaze-evoked nystagmus, oculomotor apraxia, restricted upgaze and down-drift during fixation. Primary position nystagmus was noted in seven (26%) patients. Using the ICARS scale [15], gaze-evoked nystagmus was observed in 18 (67%) patients, which was classified as transient in seven (26%), persistent but moderate in eight (30%) and persistent and severe in three (11%). Smooth pursuit was normal in five (19%) patients, clearly saccadic in 15 (56%) and slightly saccadic in seven (26%). Saccades between visual targets separated by approximately 40° were significantly hypometric in all but two patients.

### Video-Oculography (VOG)

Eye movement recordings were obtained in 29 patients. Data quality varied across tasks, with approximately one-third of recordings deemed uninterpretable due to factors such as limited task understanding or cooperation in younger children, unstable fixation or camera alignment and data quality concerns, including poor calibration or fragmented signal traces. Maintaining a steady head position was particularly challenging in patients with motor ataxia, despite the use of a chin rest or parental support. Even when calibration was adequate, the frequent presence of nystagmus made the calculation of eye velocity unreliable in pursuit tasks and prevented the calculation of pursuit gain.

#### Self-Paced Saccades

The number of self-paced saccades between two continuously presented targets executed within a 30-s interval was counted manually from the EyeSeeCam plots, a method found to be both reliable and reproducible across sessions. Performance during the 30-s self-paced saccade task was highly variable across participants. Despite verbal encouragement, many patients paused intermittently and then resumed making saccades; however, this pattern did not typically reflect a progressive slowing across the trial period. Self-paced saccade recordings were interpretable in 19 of 29 patients (65%), with saccade counts ranging from eight to 58 saccades per 30-s interval [median (Mean Absolute Deviation), 20 (8)].

#### Reflexive Saccades

Horizontal saccade recordings were interpretable in 19 of 29 participants (65%), whereas vertical saccades were interpretable in only two participants (7%). Visual inspection of the interpretable raw data plots revealed that saccadic responses were frequently hypometric, often followed by small catch-up saccades attempting to reach the target location. Manual inspection of raw traces was required to ensure that latency values reflect true saccadic responses rather than being artefacts of nystagmus.

Latency values were calculated interactively from MATLAB-generated plots from raw data of recorded horizontal saccadic responses, allowing for verification of plausibility (see Fig. 2 for an example plot). Values were then pooled across saccade amplitude and direction to derive a final quantitative outcome. Pooled saccadic latency from interpretable plots from 19 patients ranged from 174 to 498 ms (median 291 ms). Questionable calibration and prevalent nystagmus interfered with the accuracy of gain calculations, defined as the ratio of eye movement amplitude to target displacement, as the oscillations of nystagmus can either inflate or reduce the measured amplitude of the saccade. Similarly, peak velocity estimates (degrees/sec) that are derived from saccade amplitude rather than target amplitude require accurate calibration. The fast phase of nystagmus may be misclassified as part of the saccade, leading to artificially high peak velocity values.

#### Smooth Pursuits

**Fig. 3:**
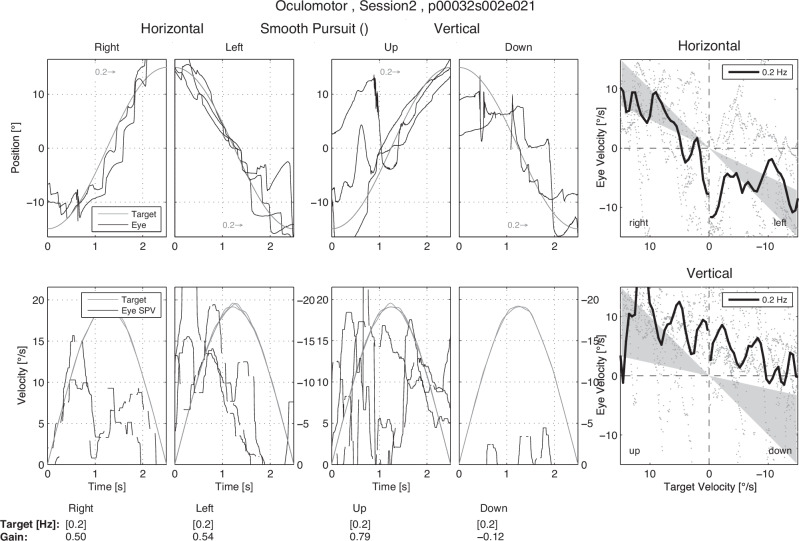
Example EyeSeeCam smooth pursuit report. Eye position and velocity traces from repeated oscillation cycles are overlaid and plotted over time (left). Phase plane plots show eye velocity versus target velocity (right), with regression slopes of black traces indicating individual smooth pursuit gains; grey areas denote normative ranges. Gain values, derived from regressions of eye and target velocity, are listed below each plot.

Smooth pursuit recordings were obtained in 26 of 29 participants (90%); however, data quality varied due to attention and inconsistent calibration. Most patients demonstrated grossly inadequate pursuit of the target that moved at rate of 0.2 Hz, characterised by frequent catch-up saccades and prolonged latency. Vertical pursuits were qualitatively more impaired than horizontal, with greater variability observed in vertical tracking. While gain is reported from EyeSeeCam reports, nystagmus can artificially inflate or reduce pursuit gain depending on its direction relative to the pursuit target (Fig. 3). The frequent presence of nystagmus within the cohort precluded confidence in pursuit gain as a reliable metric, necessitating cautious interpretation of results and preventing quantitative analysis.

#### Gaze Holding

**Fig. 4:**
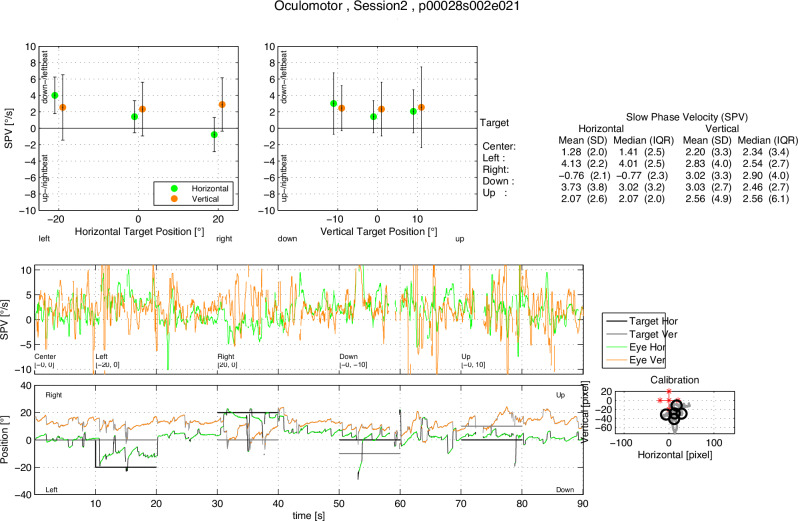
Example EyeSeeCam Gaze Holding Report showing horizontal and vertical gaze holding function, including presence of gaze holding nystagmus. Average slow-phase velocity (SPV) values for all eye positions are calculated and listed in the results table. Hor horizontal, Ver vertical.

Gaze-Holding VOG was recorded successfully in 27 participants (93%). Quantitative measures, including slow phase velocity (SPV) and interquartile range (IQR), can be extracted from the EyeSeeCam reports to assess gaze stability in both primary and eccentric gaze positions (20° right/left, 10° up/down). The internal default calibration was used to calculate the values. Elevated SPV and IQR values provide useful indicators of fixation instability and can help quantify the severity of gaze-holding deficits. Frequent fixation instability was observed across the cohort, characterised predominantly by downbeat nystagmus in both primary position and gaze-evoked conditions. Primary position vertical nystagmus, defined as SPV > 2°/s, was observed in 11 of 27 participants (41%), with seven showing downbeat and four showing upbeat nystagmus. Horizontal nystagmus in primary position was identified in six participants (22%), five of whom also exhibited significant vertical nystagmus. Substantial vertical fixation instability, defined as an IQR > 2.5°, was present in 18 of 27 participants (67%), while horizontal fixation instability was observed in 17 of 27 participants (63%). An example report from a participant with downbeat nystagmus is shown in Fig. 4. The use of default calibration, necessitated by fragmented patient calibrations, may limit the precision of these metrics and should be considered when evaluating individual and group-level findings. While individual SPV values are presented in Fig. 4 for illustrative purposes, they should be interpreted with caution. Despite these limitations, the consistent presence of downbeat nystagmus and gaze instability across recordings highlights the clinical relevance of gaze-holding assessment in A-T and supports its inclusion in future clinical studies.

## Discussion

This study provides a comprehensive profile of ophthalmic findings in A-T, highlighting the spectrum of visual function, ocular health and oculomotor abnormalities in a genetically confirmed cohort enroled in a clinical treatment trial. While visual acuity was largely preserved in most patients, significant oculomotor dysfunction was frequently observed, consistent with the neurodegenerative nature of A-T.

### Visual Function and Refractive Error

Despite the multisystem involvement of A-T, 79% of patients achieved visual acuity of 0.30 logMAR or better in their better-seeing eye, with 14% of the cohort achieving 0.10 logMAR. This aligns with previous reports suggesting that central visual acuity is often only minimally affected [3, 17].

Refractive errors were common, with low hyperopia and myopia predominating and anisometropic amblyopia present in a minority. These findings underscore the importance of routine refractive assessment and correction to optimise functional vision in this population.

### Ocular Health and Telangiectasia

Conjunctival telangiectasia was present in nearly all patients, consistent with its role as a hallmark feature of A-T. A locally designed conjunctival vascular descriptive grading approach was used, based on the number of dilated conjunctival vessels and the extent of underlying scleral obscuration. Employing this standardised classification, relatively even distribution across mild, moderate and severe presentations was found in this A-T cohort. Future work will focus on developing and validating a dedicated grading scale with improved quantitative precision.

Posterior segment imaging was feasible in most patients, although fixation instability limited acquisition, particularly in cases with significant vertical drift. Macular OCT findings were generally normal, with one case of non-specific maculopathy not attributed to A-T.

### Oculomotor Abnormalities

Eye movement recordings revealed a consistent pattern of oculomotor dysfunction, including frequent gaze instability, hypometric saccades, increased latency and poor pursuits. The hypometria and nystagmus are both consistent with cerebellar disease.

While oculomotor control may represent a useful biomarker for progression of A-T and has been shown as such for Neimann–Pick type C disease [8, 18], accurate interpretation of VOG recordings requires visual inspection of raw data to exclude invalid traces caused by calibration errors or fragmented responses, underscoring the need for cautious analysis. Despite technical challenges and variable cooperation, VOG proved feasible in most participants, yielding interpretable data for self-paced saccade count and horizontal reflexive saccade latencies in over half the cohort. While saccade gain and smooth pursuit metrics were more frequently compromised by calibration issues and frequent nystagmus, the ability to extract reliable saccade count measures and saccade latency, which are less sensitive to calibration errors, highlights the potential utility of VOG for quantifying oculomotor function.

Among the oculomotor metrics assessed, self-paced saccade count emerged as the most reliably recorded and interpretable measure in patients with A-T. This task, which requires voluntary generation of saccades between fixed targets, engages frontal cortical regions and provides insight into executive control of eye movements. Given its robustness and relative ease of administration, self-paced saccadic testing may offer a practical addition to clinical evaluations. In the current A-T cohort, 30 s self-paced saccade counts ranged from eight to 58, markedly lower than values reported in healthy young adults, where Abel and Douglas observed a mean of 69.5 $\pm$ 13.07 saccades over the same interval [19]. A low self-paced saccade count reflects impaired volitional eye movement initiation, which can hinder visual scanning, reading and interaction with dynamic environments. These deficits may contribute to slower visual responses and increased reliance on compensatory head movements, impacting daily functioning and engagement, particularly in academic and digital contexts.

Horizontal saccadic latency in the current cohort ranged from 174 to 498 ms (median 291 ms), compared to around 210 ms for 15° horizontal targets reported in a healthy normative sample by Hopf et al. [20]. This delayed initiation is consistent with dysfunction in oculomotor control pathways, particularly involving the frontal eye fields, superior colliculus and cerebellar vermis, regions known to be affected in A-T. In daily life, this may translate to slower visual responses, reduced situational awareness and increased reliance on compensatory strategies such as head movements, which themselves are often impaired by cerebellar ataxia.

Vertical saccades, when present, were slower and more variable than horizontal, further highlighting the marked vertical gaze impairment in the A-T cohort. These findings are in line with previous studies using VOG, which have described unstable vertical gaze holding, gaze-evoked nystagmus and saccadic intrusions in A-T [21]. As in Niemann–Pick type C, this may reflect the greater vulnerability of vertical saccade generation pathways to earlier impairment.

Pursuit responses in the A-T cohort were frequently fragmented, consisting of stepwise saccades rather than smooth tracking, with vertical pursuits more affected than horizontal; combined with pervasive nystagmus, this limited the reliability of gain metrics and necessitated qualitative interpretation. Pursuit gain, defined as the ratio of eye velocity to target velocity, reflects tracking accuracy; values < 1.0 indicate lagging eye movements often requiring catch-up saccades, while values > 1.0 may suggest overshooting or interference from other eye movements such as nystagmus. Further, nystagmus was common in the A-T cohort, which precluded reliable quantification of pursuit metrics.

Fixation instability and nystagmus were common during gaze holding, contributing to challenges in clinical imaging and functional vision tasks. Clinically significant nystagmus was apparent in gaze-holding plots with SPV frequently >2°/s, suggesting pathological nystagmus. High IQR values were frequently observed in the patient cohort, often exceeding 2.5–3.0°, indicating substantial fixation instability. In contrast, healthy adults typically demonstrate IQR values below 1.5–2.0° in both primary and eccentric gaze positions. This elevated variability in eye position reflects impaired gaze-holding function, likely driven by cerebellar dysfunction and the presence of nystagmus and supports the use of IQR as a quantifiable marker of oculomotor impairment in A-T [8].

### Clinical Implications

The presence of significant oculomotor dysfunction, even in the context of relatively preserved visual acuity, has important implications for daily functioning. The frequent failure of vertical saccades, reduced horizontal self-paced saccade rate and increased horizontal reflexive saccade latency are consistent with oculomotor apraxia, a recognised feature of A-T. These deficits represent a major functional limitation, contributing to difficulties with visual scanning, reading and attention, impacting daily functioning and academic performance. Patients often struggle to re-direct gaze efficiently, relying on adjunctive head movements that are further compromised by general ataxia, making accurate gaze control particularly challenging. Tasks requiring steady fixation, rapid refixation and precise visual tracking, such as reading, screen use and mobility, may be disproportionately affected. Emerging technologies such as virtual reality may be worth considering, as they offer adaptive visual environments and input methods that minimise reliance on eye movements.

The present findings suggest potential utility of oculomotor metrics, notably self-paced saccades, as biomarkers for A-T disease progression or treatment response. This study was conducted as part of a broader clinical research programme embedded within a Phase 2a/b randomised, placebo-controlled, dose-escalation trial that included eye movements as a secondary endpoint. Within this context, self-paced saccade counts demonstrated a modest dose-related improvement at 20% and 35% dosing. In contrast, changes in saccadic latency and pursuit gains were small and inconsistent across the heterogeneous cohort [13].

Future studies should explore alternative recording methods or adaptive protocols to improve data capture in younger or more severely affected patients. Eye trackers not requiring calibration, such as the Pupil Labs Neon (pupil-labs.com/products/neon), may allow for eye movement data to be collected from more severely affected individuals.

In settings where VOG is unavailable or impractical, a simple bedside method can be used to assess self-paced saccades. Patients are instructed to shift their gaze back and forth between two horizontally placed visual targets (approximately 15–20° apart) as quickly as possible over a 30-s period. The number of saccades completed is counted and qualitative observations, such as fixation accuracy, latency and reliance on head movements, are noted. This approach offers a practical, low-resource alternative for evaluating voluntary saccade generation and can be readily integrated into routine clinical assessments for individuals with A-T.

### Limitations and Future Directions

Currently, there is no published, validated scale for grading conjunctival telangiectasia in individuals with ataxia-telangiectasia. Future research aims to develop and validate a standardised assessment tool, which could enhance clinical monitoring and facilitate comparisons across studies.

Approximately one-third of eye movement recordings were uninterpretable due to poor calibration, recording quality or young patient age, limiting understanding and attention. Nonetheless, the use of recorded saccadic responses allowed for detailed quantitative analysis of latency and self-paced saccade rate, offering insights not accessible through clinical observational methods alone. In contrast, the ICARS oculomotor subscale [15], while widely used, lacks granularity, particularly in assessing fixation stability, saccadic latency and compensatory strategies. Clinical neurovisual evaluation, as described by Iodice et al. [17], offers a structured qualitative approach that is feasible in paediatric populations and captures a broader spectrum of oculomotor dysfunction. However, it remains limited by its subjective nature and lack of precision in quantifying movement parameters.

## Conclusion

This study highlights the complex ophthalmic profile of A-T, revealing that while visual acuity is often preserved, oculomotor dysfunction is both prevalent and functionally significant. Hypometric saccades, impaired smooth pursuit, gaze-evoked nystagmus and fixation instability were common and quantifiable using non-invasive eye movement recordings in the majority of patients. These findings underscore the importance of integrating detailed oculomotor assessment into routine ophthalmic care for A-T patients.

## Data Availability

No datasets were generated or analysed during the current study.
